# Challenges in Protocol Development and Interpretation of the Schistosomiasis Consortium for Operational Research and Evaluation Intervention Studies

**DOI:** 10.4269/ajtmh.19-0805

**Published:** 2020-05-12

**Authors:** Charles H. King, Nupur Kittur, Ryan E. Wiegand, Ye Shen, Yang Ge, Christopher C. Whalen, Carl H. Campbell, Jan Hattendorf, Sue Binder

**Affiliations:** 1 Center for Global Health and Diseases, Case Western Reserve University, Cleveland, Ohio;; 2 Schistosomiasis Consortium for Operational Research and Evaluation, Center for Tropical and Emerging Global Diseases, University of Georgia, Athens, Georgia;; 3 Parasitic Diseases Branch, Division of Parasitic Diseases and Malaria, Centers for Disease Control and Prevention, Atlanta, Georgia;; 4 Swiss Tropical and Public Health Institute, Basel, Switzerland;; 5 University of Basel, Basel, Switzerland;; 6 Department of Epidemiology & Biostatistics, University of Georgia, Athens, Georgia

## Abstract

In 2010, the Schistosomiasis Consortium for Operational Research and Evaluation (SCORE) began the design of randomized controlled trials to compare different strategies for praziquantel mass drug administration, whether for gaining or sustaining control of schistosomiasis or for approaching local elimination of *Schistosoma* transmission. The goal of this operational research was to expand the evidence base for policy-making for regional and national control of schistosomiasis in sub-Saharan Africa. Over the 10-year period of its research programs, as SCORE operational research projects were implemented, their scope and scale posed important challenges in terms of research performance and the final interpretation of their results. The SCORE projects yielded valuable data on program-level effectiveness and strengths and weaknesses in performance, but in most of the trials, a greater-than-expected variation in community-level responses to assigned schedules of mass drug administration meant that identification of a dominant control strategy was not possible. This article critically reviews the impact of SCORE’s cluster randomized study design on performance and interpretation of large-scale operational research such as ours.

## OVERVIEW

In performing its mission to inform program managers and policy-makers working in schistosomiasis control, the Schistosomiasis Consortium for Operational Research and Evaluation (SCORE) project confronted a number of opportunities and challenges in the design, performance, and analysis of its large field research studies that were meant to determine implementation effectiveness in real-world settings.^1^

Operational research has been defined as “…the search for knowledge on interventions, strategies, or tools that can enhance the quality, effectiveness, or coverage of programs in which the research is being done”^2^ and, as such, is often quite different in design and performance settings from those used in standard clinical trials. In designing healthcare research, investigators often need to choose between narrowly focused, highly controlled studies that can provide clear, explanatory answers about treatment efficacy in a nearly ideal setting versus pragmatic operational studies that allow for real-world variation.^3^ For SCORE’s objectives, this latter type of study was expected to be better suited to show how an intervention would perform in general practice.^3–5^

Interventional operational research projects are often “pragmatic trials,” aiming to determine effectiveness at the program-level scale in the intended implementation setting. During the course of large-scale interventions, previously unknown heterogeneities in treatment efficacy, side-effects, and overall effectiveness are likely to be uncovered. Moreover, program implementation may not be consistent: ministries of health may reorganize health priorities and reallocate resources or experience periodic shortfalls in drugs, diagnostics tests, or personnel. These effects need to be identified, quantified, and documented, as this knowledge is necessary for programs to continue to improve.^2,6^

The objectives of the large-scale SCORE operational research intervention studies were as follows:

1. To identify optimal schedules and target populations for mass delivery of praziquantel for control of *Schistosoma haematobium* and *Schistosoma mansoni* infections in endemic areas of sub-Saharan Africa.^1^ For SCORE, there were seven randomized trials that were termed the gaining and sustaining control studies. Two levels of baseline schistosomiasis prevalence were separately studied. The gaining control studies in Kenya, Mozambique, Niger, and Tanzania focused on communities with baseline school-age prevalence ≥ 25%; the sustaining control studies in Côte d’Ivoire, Kenya, and Niger focused on communities with school-age prevalence between 10% and 24% (see King et al.^7^ in this issue for details).
2. To determine the potential benefits of mass drug administration (MDA) on *Schistosoma* infection–associated morbidity and to compare the relative impact of every-year treatment (given community-wide) to that of a standard biennial school-based treatment program. Nested cohort studies of morbidity were, therefore, embedded within gaining control studies in Kenya, Mozambique, Niger, and Tanzania for this purpose.^8^ (SCORE termed these the cohort morbidity studies; see King et al.^9^ in this issue for details.)
3. To identify the ability of more intensive (twice yearly) schedules of drug delivery, given alone or in combination with local snail control or with behavior change interventions, to approach elimination of transmission in a region of Zanzibar that had successfully reduced local *Schistosoma* transmission to very low levels.^10^ (See Campbell et al.^11^ in this issue for details of the SCORE Zanzibar elimination study.)
4. To study the impact of different schedules of repeated MDA, with or without supplemental snail control, on rates of infection in areas where transmission is highly seasonal. This SCORE Seasonal elimination study was performed in *S. haematobium*–endemic communities in northern Cote d’Ivoire. The trial compared the effect of annual MDA timed to be given either before or after the seasonal rainfall period. Those two study arms were also compared to an arm given biannual MDA (given *both* before and after seasonal transmission) and to an arm where communities received annual pre-rainfall MDA combined with supplemental thrice-yearly local snail control.^12^

These 13 studies were performed in six different countries and involved hundreds of thousands of study participants who were tested in hundreds of locations over multiple years, often in resource-limited settings. An undertaking of this scale raised complex issues for implementation, supervision, data quality, and analysis of study findings. This article provides a critical review of the issues of study design and interpretation for such large-scale operational research. Recommendations for future studies are offered, with a more detailed list provided in Binder et al.^13^ in this supplement.

## STUDY DESIGN CHALLENGES FOR SCORE’S RANDOMIZED PRAGMATIC TRIALS

The development process for the gaining and sustaining field trials began in 2010 at SCORE-sponsored meetings of experienced schistosomiasis field researchers and program managers and other neglected tropical disease (NTD) experts (see Colley et al.^14^ in this issue). The target questions identified at these development meetings were as follows: 1) Is there different MDA impact with different frequencies of treatment implementation (annual, biennial, or 2 years on then 2 years off)? 2) Is there a different MDA impact based on the population targeted for participation in MDA (i.e., school-age children [SAC] treatment only, delivered in a school-based treatment [SBT] program or SAC plus adult treatment delivered in a community-wide treatment [CWT] program)? 3) What is the impact of non-adherence to MDA? In addition to these objectives, collection of non-research–related program cost and performance data were planned to estimate the incremental cost-effectiveness of more intensive interventions.

Schistosomiasis Consortium for Operational Research and Evaluation chose a study design that randomly assigned communities to different MDA schedules and to either SBT or CWT approaches. Community eligibility for participation was determined by preliminary screening surveys of community prevalence of *S. haematobium* or *S. mansoni* among 13- to 14-year-olds. Community-level randomization was chosen for SCORE randomized trials because environmental changes in local transmission related to reduced egg contamination might occur if high-coverage mass treatment resulted in reduced local *Schistosoma* exposure. This, in turn, could result in an indirect effect on long-term infection prevalence and intensity outcomes.^15,16^ Very similar issues accompany operational research trials of new vaccines, where herd immunity effects can impact the apparent efficacy of vaccine delivery.^17^

Hence, to evaluate the group-level benefits of MDA intervention, randomization at the community level was chosen for SCORE operational research studies. In addition, this cluster randomized design focused at the community level could be integrated more easily within the planned activities of national control programs for schistosomiasis (and other NTDs) than would a study requiring individual-level intervention and assessment. The results of this type of trial were expected to be more useful to ministries of health in deciding which method of MDA was likely to be most effective for controlling schistosomiasis morbidity in their population.

### Impact of cluster randomization on SCORE results.

Cluster randomized field trials have inherent limitations, some of which were encountered in the SCORE research studies. One limitation is that potential confounding factors such as daily population movement and local access to safe water sources may not be equally distributed among the different assigned intervention groups, especially if the units within a group already differ with regard to the planned study outcome metric at baseline.^18,19^ Moreover, as in any randomized study, some units may not reliably receive their assigned treatment intervention. It is possible that regardless of the quality of randomization, unbeknownst to the research team, study participants may privately receive the intervention through sources other than the trial itself. In SCORE field studies, treatment allocation could not be concealed from the study participants or the intervention teams. For these reasons, there was risk of unintended bias in performance of the SCORE studies.^1,10,19,20^

As an example, we performed a sensitivity analysis to compare the results of the SCORE cohort studies’ basic intention-to-treat analysis against a marginal structural model analysis^21,22^ to assess the effect of the lack of balance of community-level factors between arms in one of the SCORE trials. In our basic intention-to-treat analysis, the study units were analyzed according to the intervention they were assigned, regardless of what intervention they actually received.^22^ By contrast, the marginal structural analysis used the observed probability of receiving the study treatment to weight a regression model that estimates the population average, or the marginal, causal effect of implementing the assigned the intervention as it actually occurred.

**Figure 1.**
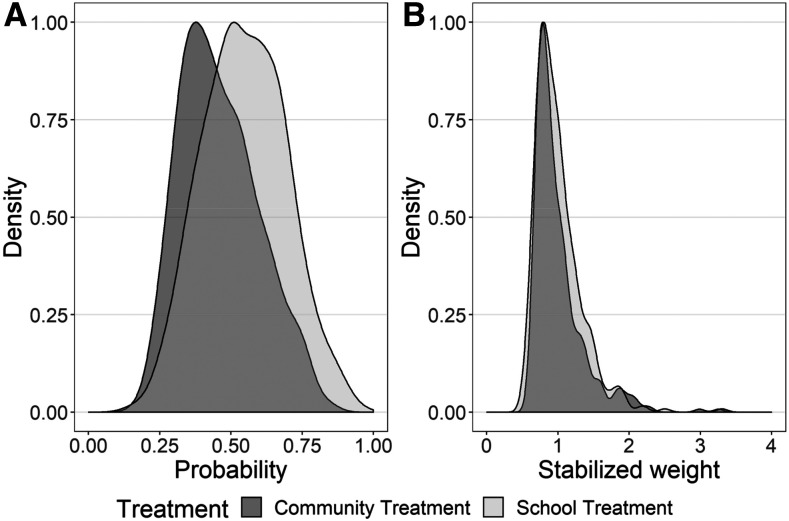
Results of a marginal structural reanalysis of Schistosomiasis Consortium for Operational Research and Evaluation data from the Kenya morbidity cohort study. (**A**) Children’s reduced probability of treatment in the community-based treatment area. (**B**) Better comparability for the two study arms with the use of weighted adjustment of treatment inputs.

Unfortunately, this approach could not resolve the challenge of community-level differences in response to intervention. It was clear that cluster-based randomization did not achieve a balance of known and unknown confounding factors between these two implementation study arms. Even in the analysis of the SCORE nested cohort morbidity study in Kenya,^8,23^ which had the greatest number of possible inputs regarding individual- and community-level influences, a statistically significant difference in infection-related study outcomes could not be established between the every-year CWT study arm and the every-other-year SBT arm. As can be seen in Figure 1A, the two study arms, as implemented, did not provide local children with an equal probability of treatment. The peak likelihood of getting treated in the community-based study arm was about 40%, instead of the expected 50%. After applying a weighted analysis (using the inverse probability of receiving treatment in each arm),^22^ a better effective balance of CWT and SBT inputs was achieved for comparison (Figure 1B). In the unweighted analysis, the calculated odds of infection at the end of the study was 26% higher among the communities assigned to SBT (odds ratio [OR] = 1.26, 95% CI: 0.42, 3.77) than in communities assigned to CWT. When the analysis was redone using the weighted marginal structural model, the recalculated odds of infection at the end of the study period were 63% higher in SBT versus CWT communities (OR = 1.63, 95% CI: 0.37, 7.21), suggesting a potentially large difference in outcomes based on the frequency and age-group coverage of the MDA. However, for both types of analysis, because there was a great deal of variability among the responses of individual communities, the CI for these ORs include 1, meaning we cannot conclude with statistical confidence that one intervention arm is preferable, in general, over the other.

Because the OR estimate changed with adjustment for confounders in the marginal structural model, the reanalysis indicated that randomization of a relatively limited number of communities (25 per group) also did not achieve an adequate balance of other confounders between the two study arms. A larger sample size, with fewer study intervention arms may have avoided this problem.^24^ It has been suggested that an initial re-randomization to obtain better balance in known risk factors might have allowed the 25 villages/arm study design to provide more convincing evidence of a differences in endpoint outcomes among the study arms. However, the information available on community-level factors was relatively limited at the time of randomization at the beginning of the SCORE studies. Schistosomiasis Consortium for Operational Research and Evaluation has since performed post hoc analysis of community-level risk factors in some depth, including assessment of the effects of starting prevalence, treatment compliance, local water supply, hygiene factors, snail abundance, road access, landscape use, rainfall, and other factors, but have not found any consistent population-specific or environmental factors linked to local risk of persistent high transmission in our study communities.^25–28^ Because heterogeneity in community-level response to treatment is not easily predictable on this basis, it is not clear how re-randomization could have been performed or whether it would have ultimately been effective.

### Determination of study size.

The SCORE study design effort was based on the desire to compare the relative of benefits of alternative MDA intervention schedules (as recommended previously by different schistosomiasis control experts) in terms of their community-level impact on *Schistosoma* infection prevalence and intensity among school-age children. To insure a more general applicability of the results, we also aimed to include a broad-based representation of *Schistosoma*-endemic countries in sub-Saharan Africa. Trade-offs in design were necessary because of budgetary limits and constraints in SCORE partners’ capacity to perform the necessary work on a large scale.^13^ In performing the power analysis for the planned operational research trials, it was clear that the use of community as an implementation unit constrained statistical power and would entail a necessary adjustment of the uncertainty in impact estimates to account for community intra-class correlation effects.^1^

The SCORE researchers did have a priori knowledge of the range of community- or district-level prevalences based on past mapping surveys, but there were few data available about what to expect in terms of the likely variance in community-level infection prevalence and mean infection intensity following multiple rounds of MDA in a program setting. When harmonizing the gaining and sustaining implementation protocols among the chosen country teams, we assumed a potential variance in prevalence outcomes of ∼20%. This supported the choice of using 25 communities per treatment study arm, with sampling of 100 9-to-12-year-old children per community each year. In the final analysis, based on our formalized statistical analysis plan, the variance in community-level responses turned out to be substantially greater than expected,^24,29^ which ultimately limited our ability to claim clear-cut advantages to any given implementation strategy (see Kittur et al.^30^ in this issue regarding the problem of “persistent hotspots”). Because of this large variance and the limited advance information on potential confounders, our ability to stratify communities by risk and our power to detect statistically significant differences in infection and morbidity outcomes was more limited than we had anticipated.^8^ In retrospect, the use of fewer study arms in each trial, with inclusion of a greater number of community units per arm, might have overcome these limitations of our design, but would have decreased the number of questions that could have been asked.

The SCORE study that focused on approaches for *S. haematobium* elimination in Zanzibar provides an example of the kinds of trade-off that had to be made related to sample size in the context of a national program. Communities were randomized in a 1:1:1 ratio to one of three different intervention arms: 1) twice-yearly MDA with praziquantel, 2) twice-yearly MDA combined with focal snail control, or 3) twice-yearly MDA combined with behavior change interventions for children, teachers, and adults in affected communities.^10^ In performing power analysis for design of the study, it was found that to reach a desired power of 80%, the number of community clusters (shehias) that would be needed would exceed the total number of *S. haematobium*–endemic shehias on the islands. In addition, the desired sample size of participants per shehia was not logistically practicable. Therefore, the choice of 15 shehias/intervention arm/island and the number of subjects tested each year was a compromise between what was considered optimal and what was practically achievable.^31^

Despite this problem, there were strong advantages to using the Zanzibar location. Zanzibar was a clearly defined geographic area with strong political commitment and a long history of having implemented effective praziquantel MDA program. The suboptimal study size meant that the quality of evidence for the randomized comparison would be graded as “low” because of the limitations in the design. However, its results now provide extensive “pilot” data for the design of any subsequent comparison studies. Like other complex intervention trials, it also provides extensive observational data about the challenges for implementation of combined interventions for effective schistosomiasis control.^3,5^ Ultimately, the study in Zanzibar clearly demonstrated that prevalence and intensity can be reduced even in areas that have achieved WHO criteria for elimination as a public health problem, but it also established that interruption of transmission will be difficult, and unlikely to occur with MDA alone.^13^

For future studies where the focus is on elimination, the expected starting and ending prevalences will be, by definition, very low, which poses additional challenges. The absolute difference in prevalences between trial arms will be small, which increases the required sample size dramatically. In addition, many of the clusters will show an apparent prevalence of zero, which might compromise the validity of routinely used statistical methods such as random effect models. Furthermore, diagnostic test performance becomes critical, and imperfect tests will bias the results toward the null. Even if the test specificity is 99%, the apparent prevalence will not fall below 1% even if the disease is truly eliminated. Therefore, prevalence might be not a suitable outcome for randomized trials in very low-prevalence settings, and there is currently no agreement about appropriate alternatives. Simulation studies of elimination surveillance suggest an optimal number of participants per cluster of at least 200,^32^ but testing this number in each cluster may not prove feasible in the field.

### Potential threats to validity in SCORE implementation studies.

The operational research approach of SCORE treatment studies meant that sites had flexibility in how the interventions were delivered. Schistosomiasis Consortium for Operational Research and Evaluation treatment delivery was meant to emulate the typical application of treatment interventions in national programs. In some cases, the SCORE project directly used national program teams for praziquantel delivery following the assigned SCORE schedules. In other locations, SCORE research teams, in close collaboration with national NTD control partners, separately provided praziquantel treatment in participating communities. Although SCORE investigators were experienced in conducting field studies and had participated in the development of harmonized SCORE protocols for study performance, potentially influential protocol deviations did occur in several locations. Examples are provided in Table 1. A more detailed discussion of these issues is found in two articles by Binder and others^13,33^ in this issue.

**Table 1:** Examples of potentially influential protocol deviations experienced during SCORE study implementation

Location	Challenge	Impact
Niger	Randomization by region and not by community	This prompted complete revision of the Niger study protocols; exclusion from main SCORE analysis
Kenya and Tanzania gaining control studies	Decision not to use schools as a venue within community-based treatment arms in years 1 and 2	Lower than desired coverage of school-age children in enrolled villages receiving community-wide treatment in Kenya and Tanzania in years 1 and 2
Mozambique gaining control study	Allocation of community drug distributors was not done based on the size of the population that needed to be reached, and supervision was minimal	MDA coverage was suboptimal in many communities
All	Delays in data inputs and data cleaning, uneven formats for reporting	Late detection of implementation problems; inability to provide well-timed correction of coverage errors
Tanzania, Mozambique, Niger, Cote d’Ivoire	Difficulty categorizing costs and separating MDA costs from other costs; nonuniform reporting of program vs. research costs	Inability to develop summary estimates of programmatic cost-effectiveness across all SCORE studies, except for Kenya’s gaining control study

MDA = mass drug administration; SCORE = Schistosomiasis Consortium for Operational Research and Evaluation.

Across the Kenya, Niger, and Tanzania locations, seven of the 600 communities were inadvertently treated twice with praziquantel in the same year. In Kenya and Niger, some praziquantel treatments were given in years that were intended to be praziquantel drug holiday years. Off-schedule (not truly annual or biennial) treatment rounds and surveys also occurred because of weather-related delays and other issues (see following paragraphs).

Some projects did not reach the planned SAC coverage targets and did not follow up with the agreed-on mop-up delivery visits, resulting in undertreatment. In other communities, reported SAC coverage, based on available population numbers, was > 100%. Among the possible explanations is participation of people traveling from surrounding communities who also sought treatment. This may have yielded contamination of treatment assignment in those neighboring communities during the study period, potentially decreasing the detection of differences between assigned intervention arms. On Zanzibar, the contiguous borders of participating shehias randomized to different study arms raised the possibility of overlap of treatment delivery, particularly for areas adjacent to those receiving snail control interventions. Shared watersheds created the possibility that environmental snail control would impact more than one location. Enrolment in schools away from a home shehia may also have exposed children to the behavior change teaching outside of their shehia’s assigned intervention.

Other factors that arose during the operational trials were those that could modify local participation of community residents or the timing of drug delivery and follow-up surveys for prevalence and intensity of infection. These included local and national elections and election-related violence, extreme weather events (2015 EL Niño rains in East Africa), intercurrent bacterial epidemics, and annual periods of food scarcity before harvest or sporadic food shortages due to drought, which may have led to decreased drug uptake because of fear of gastrointestinal side effects from praziquantel taken on an empty stomach.

### Timing of final impact assessment surveys to compare twice-yearly versus annual treatments.

In many endemic locations, transmission of schistosomes is not uniform throughout the year. Such seasonality means that the timing of impact assessments may matter greatly in terms of gauging the size of treatment effects. Where transmission is strongly seasonal, assessment surveys performed at any time during the non-transmission period are expected to be roughly equivalent because of the years-long lifespan of established, egg-producing adult worms. However, where transmission is continuous and new infections continue to accumulate over time, the time between last treatment and testing is very likely to affect the estimate of posttreatment infection prevalence and intensity.

This issue posed a problem following the modification of the Niger *S. haematobium* gaining and sustaining studies to annual versus biannual treatment arms.^7^ Infection outcomes were measured in year 5, after 4 years of participation. However, this meant that some communities were assessed 6 months after their last treatment (the twice-yearly communities), whereas others were assessed 12 months after their last treatment. In the subsequent Cote d’Ivoire study of seasonally timed drug delivery,^12^ evaluation was performed in two ways: 1) infection prevalence a full year after the final scheduled annual or biannual MDA given in each arm and 2) infection prevalence for all arms at a single time period (December 2018), which was 12 months after the last annual dose and 8 months after the last biannual doses.

## SUMMARY

There were both strengths and weaknesses to the SCORE operational research approach. Irregularities in performance of these studies, which involved 620,000 participants in 860 communities, did occur, which may have reduced our ability to detect real differences between the assigned treatment schedules and age-group coverages in the different study arms. An important finding that did emerge from the SCORE trials included the fact that although all interventions significantly reduced mean infection prevalence and intensity, the wide variability in community responses (the “persistent hotspot” problem^29^) poses a clear challenge to the effectiveness of a blanket application of the current WHO schistosomiasis control recommendations.^34,35^

Cluster randomization by community and a wide variation in response to MDA posed challenges in identifying statistically significant differences among our treatment intervention study arms. Given appropriate caveats in performing post hoc secondary analysis, it does appear that four rounds of treatment over 5 years offers advantages over two rounds of treatment in terms of reducing SAC infection prevalence and/or intensity in higher prevalence *S. haematobium*– and *S. mansoni–*endemic communities.^24,36,37^ Similarly, after post hoc adjustment for starting prevalence, 4 years of CWT appears likely to yield better control of *S. mansoni–*related morbidity than every-other-year SBT.^37^ Addition of snail control or structured behavior change intervention may also improve outcomes beyond those obtained with intensive (biannual) MDA alone.^31^ Last, in the gaining control studies among Kenyan and Mozambican communities that received only SBT, there appeared to be a reduction in local force of transmission as reflected in reduced rates of infection among local adults and preschool children at the endpoint of those studies.^7^ This phenomenon was not observed in the other SCORE locations, however.

These preliminary exploratory findings based on established SCORE data will help define the future operational research agenda for schistosomiasis control.^38^ Those next-generation projects are expected to further refine knowledge of the diverse operational aspects of control, with improved program performance, which could ultimately lead to elimination of *Schistosoma*-related disease.
